# Ameliorative potential of dietary supplements, ZnO-K, citrus essential oil, and pumpkin seed oil, on sperm quality in Nile tilapia: Insights from CASA, DNA integrity, antioxidant enzymes, and gene expressions

**DOI:** 10.1007/s10695-025-01529-4

**Published:** 2025-06-23

**Authors:** Marwa M. Ali, Kamal Fathy Elboray, Engy T. Megahed, Hany T. Abu-Taleb, Alshimaa E. Elsayed, Eman Y. Mohammady, Mona S. Amer, Soliman A. Morsi, Eman M. Abbas, Mohamed S. Hassaan, Hosam Elsaied

**Affiliations:** 1 Department of Animal Production, Fish Research Laboratory, Faculty of Agriculture, Benha University, Benha, 13736 Egypt; 2 Department of Fish Reproduction, National Institute of Oceanography and Fisheries, NIOF, Cairo, Egypt; 3 Department of Genetics and Genetic Engineering, National Institute of Oceanography and Fisheries, NIOF, Cairo, Egypt; 4 Department of Fish Nutrition, National Institute of Oceanography and Fisheries, NIOF, Cairo, Egypt

**Keywords:** Antioxidant supplements, CASA parameters, DNA integrity, Enzyme bioassay, Gene expression, *O.* niloticus, Sperm quality

## Abstract

Improving sperm quality is essential for enhancing the reproductive efficiency of *Oreochromis niloticus*. This study evaluated the effect of three dietary antioxidant supplements, kaolinite-doped zinc oxide (ZnO-K), citrus essential oil (CEO), and pumpkin seed oil (PSO), on sperm quality parameters. A suite of integrative assessments, including Computer-Assisted Semen Analysis (CASA) parameters, spermatozoa DNA integrity, antioxidant enzyme assays, and gene expression profiling, was applied to validate sperm quality. A total of 192 adult males (mean weight 421.31 g ± 30.41 SD) were assigned to four dietary groups, each with three replicates. The control group received a basal diet without supplementation. The experimental groups were fed diets supplemented with ZnO-K (0.06 g kg⁻^1^), CEO (10 g kg⁻^1^), or PSO (15 g kg⁻^1^). ZnO-K supplementation significantly (*P* < 0.05) increased milt volume (1.40 ± 0.10 ml) and sperm concentration (5.676 × 10^9^ sperm ml^−1^), along with marked improvements in CASA parameters (sperm motility and velocity) and DNA integrity. Antioxidant enzymes, catalase, CAT, glutathione peroxidase, GPX, and superoxide dismutase, SOD, were also significantly elevated in the ZnO-K group, recording activity values of 47.333 ± 1.452 U ml^−1^ milt, 65.667 ± 5.547 mU ml^−1^ milt and 60.667 ± 3.382 U ml^−1^ milt, respectively. Gene expression analysis revealed that CEO induced the highest upregulation of *HSP70*, while both CEO and ZnO-K significantly upregulated *CC chemokine* expression compared to control. In contrast, PSO consistently showed the lowest performance across all measured parameters. Collectively, ZnO-K supplementation most effectively enhanced sperm physiological and biochemical quality, whereas CEO predominantly supported cellular stress protection through gene expression modulation. These findings offered practical implications for optimizing artificial reproduction and sperm cryopreservation strategies in tilapia aquaculture.

**Supplementary Information:**

The online version contains supplementary material available at 10.1007/s10695-025-01529-4.

## Introduction

The sustainability of *O. niloticus* breeding programs relies heavily on sperm quality, a primary determinant of fertilization success and overall reproductive efficiency. Oxidative stress, resulting from an imbalance between reactive oxygen species (ROS) and antioxidant defenses, negatively impacts sperm motility, viability, and DNA integrity (Cabrita et al. 2014). Thus, enhancing sperm quality through antioxidant-enriched dietary supplementation is vital for sustainable *O. niloticus* production (Sarmento et al. 2017; Bombardelli et al. 2023).

Optimizing feed utilization in male *O. niloticus* not only promotes somatic growth but also improves reproductive parameters, including sperm quality. These enhancements are achieved through the modulation of blood and serum biochemical markers, favorable changes in morphometric indices such as the gonadosomatic index (GSI) and hepatosomatic index (HSI), enriched fatty acid profiles, and sufficient mineral content, all of which are integral to successful aquaculture practices (Engdaw and Geremew 2024; Kabir et al. 2024). Nutritional antioxidants are particularly effective in improving sperm motility, DNA stability, antioxidant enzyme activity, and the expression of stress-resistance-related genes in aquatic organisms (Ciereszko and Dabrowski 2000; Gammanpila et al. 2007; Zhang et al. 2015; Mahanty et al. 2017). ZnO-K has shown antioxidant activity, efficacy at 90 mg kg^−1^ diet, in enhancing biological parameters, reproductive indices, lipid profiles, and antioxidant enzyme activities in *O. niloticus* (Soaudy et al. 2021). However, previous studies on ZnO-K have been limited by their reliance on manual assessments of semen quality of Nile tilapia, omitting key parameters such as sperm velocity and DNA integrity, critical indicators of sperm function (Soaudy et al. 2021). Although studies on CEO in *O. niloticus* are scarce, lemongrass-derived essential oils have demonstrated positive effects on tilapia growth and antioxidant status (Al‐Sagheer et al. 2018), while citrus lemon extracts have improved sperm quality in mice (Rahayu and Hanizar 2021) and overall health in *O. niloticus* (Mohammady et al. 2021). PSO, rich in polyunsaturated fatty acids, flavonoids, and antioxidants, has shown efficacy in enhancing sperm quality in rats by mitigating oxidative stress (Benalia et al. 2015; Aghaie et al. 2016). Despite its promising properties, the effect of PSO on fish sperm quality remains unexplored, with existing studies focusing solely on growth performance in fish (Dada and Ejete-Iroh 2015; Greiling et al. 2018).

Advanced methodologies are essential for accurately evaluating fish sperm quality. CASA provides precise measurements of motility parameters such as curvilinear velocity (VCL), straight line velocity (VSL), and average path velocity (VAP), across various fish species (Rurangwa et al. 2001; Caldeira et al. 2019). While still limited in *O. niloticus*, DNA integrity assays have been successfully applied in other fish species such as zebrafish and Arctic charr (Gosálvez et al. 2014; Jeuthe et al. 2022).

Antioxidant enzymes, CAT, GPX, and SOD, play pivotal roles in neutralizing oxidative damage, thereby maintaining sperm structure and functionality (Drevet 2006; Agarwal et al. 2014; Perumal 2014). Gene expression analysis also offers valuable insights into the molecular mechanisms that regulate sperm function and response to oxidative stress. Specifically, the genes *HSP70* and *CC chemokine* are crucial in stress response pathways (Caballero-Campo et al. 2014; Liu et al. 2019). *HSP70* is a molecular chaperone involved in protecting cells from apoptosis and is critical for cellular resistance and stress adaptation (Ferraz et al. 2019). *HSP70* expression is strongly correlated with sperm motility and reproductive success (Solanki et al. 2023). *HSP70* is upregulated in response to ROS accumulation, aiding in protein refolding, preventing aggregation, and stabilizing mitochondrial function (Feder and Hofmann 1999). Similarly, *CC chemokines*, known for their immunomodulatory roles, are implicated in reproductive biology (Palomino and Marti 2015). *CC chemokines* help recruit immune cells to the gonadal environment, promoting the removal of damaged cells and supporting testicular homeostasis (Zlotnik and Yoshie 2012). Antioxidants may influence *CC chemokine* expression, highlighting a cross-talk between redox signaling and immune function during spermatogenesis (Bansal and Bilaspuri 2011). Elevated antioxidant enzyme activity, as observed with dietary phytogenic supplementation, often correlates with increased *HSP70* expression, indicating a coordinated cellular strategy to mitigate oxidative injury in sperm cells (Pasri et al. 2024). The upregulation of *HSP70* and *CC chemokine* in response to enhanced antioxidant defense represents an adaptive protective mechanism designed to maintain sperm integrity under oxidative stress. This study marks a significant advancement in the molecular understanding of fish reproduction, as it is the first to investigate *HSP70* and *CC chemokine* expressions in *O. niloticus* sperm.

Despite the demonstrated benefits of antioxidant supplementation on the reproductive performance of *O. niloticus* (Sarmento et al. 2017; Hassona et al. 2020; Soaudy et al. 2021; Bombardelli et al. 2023), limited attention has been given to their direct impact on sperm quality. Moreover, sperm quality evaluations have often relied on manual assessments focused narrowly on motility, lacking methodological standardization and omitting key physiological and molecular indicators. This study aims to evaluate the potential of ZnO-K, CEO, and PSO as dietary supplements to enhance sperm quality in *O. niloticus*, utilizing a holistic and integrative approach. Advanced sperm quality assessment tools, including CASA parameters, DNA integrity assay, antioxidant enzyme analyses, and gene expression profiling, were employed. This comprehensive approach is expected to provide a robust, multifactorial assessment of sperm quality in response to dietary supplementation, thereby filling critical knowledge gaps in sperm examination and introducing qualified tilapia sperm for artificial fertilization and cryopreservation.

## Materials and methods

### Fish management and experimental design

Wild male broodstock of *O*. *niloticus*, which feed on planktonic organisms, were collected from Lake Nasser, Egypt, one of the largest habitats for this species (Elsaied et al. 2019). The males were transported to the nearby aquaculture research station, the National Institute of Oceanography and Fisheries, NIOF, Egypt. A total of 192 adult males were selected for the study, with a mean total length of 27.15 cm ± 2.45 SD and a mean body weight of 421.31 g ± 30.41 SD. The selected males exhibited a gonadosomatic index (GSI) ranging from 1.5% to 2.5%. Males were assigned to four groups, each with three replicates, 16 individuals per replicate, to enhance the results'robustness (Soaudy et al. 2021). The groups were evenly distributed among indoor concrete ponds, each measuring 4.50 m in length, 2.25 m in width, and 0.75 m in height. During the experimental period, water temperature was maintained between 25 °C and 27 °C. Key water quality parameters were consistently monitored and maintained as follows: dissolved oxygen at 6.0 ± 0.03 mg l^−1^, total ammonia at 0.15 ± 0.02 mg l^−1^, nitrite (NO_2_) at 0.02 ± 0.004 mg l^−1^, and nitrate (NO_3_) at 0.74 ± 0.06 mg l^−1^. All physicochemical water parameters were monitored in accordance with standard methods APHA, AWWA and WPCF (1989).

### Experimental diets

Four experimental diets were formulated (Table 1). Diet 1 served as the basal control without supplementation. Diet 2 was supplemented with 0.06 g kg^−1^ ZnO-K synthesized via a hydrothermal method with slight modifications from the protocols described by Mohammady et al. (2021), Hrenovic et al. (2012), and Soaudy et al. (2021). Pure CEO and PSO were purchased from local markets in Egypt, and their chemical compositions were provided in Supplementary Tables 1 and 2, respectively. Diet 3 was supplemented with 10 g kg^−1^ CEO (Kesbiç et al. 2020). Diet 4 included 15 g kg^−1^ PSO (Dada and Ejete-Iroh 2015). Each of ZnO-K, CEO, and PSO was mixed thoroughly with other ingredients in the basal diet (350 g kg^−1^ crude protein) (Table 1), using a commercial feed mixer (Hobart Corporation, Troy, OH, USA) and blended with soybean oil. Distilled water was added to the mixture to form a dough, which was then extruded through a hand-operated noodle maker. The pellets were dried at room temperature, sealed in cellophane bags, and stored at − 4 °C until use. Gross energy was calculated based on the method of Brett (1973), and proximate composition was analyzed according to AOAC (2012). Male fish were fed the experimental diets at a rate of 3% of their body weight, administered twice daily for a period of 60 days (Vilela et al. 2003).

**Table 1:** Ingredients and proximate composition of the basal diet (g/kg diet)

Ingredients	Basal diet
Fish meal	150
Soybean meal	540
Yellow corn	120
Wheat bran	120
Soybean oil	40
Zinc-free premix^a^	30
*Proximate analysis*	
Dry matter	895.5
Crude protein	350
Crude lipid	72.0
Ash	49.2
Fiber content	53.3
NFE^b^	52.94
GE^c^ (kJ/g dry matter)	20.19

^a^ Vitamin and mineral mix (mg or g/Kg diet): MnSO4, 40 mg; MgO, 10 mg; K2SO4, 40 mg; KI, 0.4 mg; CuSO4, 12 mg; Ferric citrate, 250 mg; Na2SeO3, 0.24 mg; Co, 0.2 mg; retinol, 40,000 IU; cholecalciferol, 4000 IU; α-tocopherolacetate, 400 mg; menadione, 12 mg; thiamine, 30 mg; riboflavin, 40 mg; pyridoxine, 30 mg; cyanocobalamin, 80 mcg;;nicotinic acid, 300 mg; folic acid, 10 mg; biotin, 3 mg; pantothenic acid, 100 mg; inositol, 500 mg; ascorbic acid, 500 mg. 2*B. acidophullus* was prepared to obtain (1.47 × 107 CFU kg- 1 approximately. ^b^ NFE (Nitrogen free extract) = 100- (crude protein + lipid + ash + fibre content). ^c^ Gross energy, calculated using gross calorific values of 23.63, 39.52 and 17.15 kJ g^−1^ for protein, fat and carbohydrate

### Collection of fish milt

A milt sample was collected from each of the 16 males per replicate by gently applying pressure to the abdomen, avoiding contamination with water, feces, or urine, according to Abascal et al. (2007). Milt was collected directly into sterile, pre-chilled Eppendorf tubes and maintained on ice. All analyses were conducted immediately after sampling to ensure sample integrity.

### CASA parameter measurements

Sperm motility and velocity parameters were assessed using CASA system (Spermolyzer, Mira Lab, https://mira-lab.com/product/spermolyzer/), equipped with a high-speed digital camera and software optimized for fish sperm analysis. The system was configured to capture 30 frames per second, and 400–700 individual spermatozoa were analyzed per sample. CASA setting was applied according to the methodology of Alcántar‐Vázquez et al. (2022).

### Sperm motility and velocity measurements

Sperm motility and velocity were evaluated using the CASA system. A fresh milt sample of approximately 20 µl was taken from the total milt volume of each male (Table 2) in each replicate and immediately diluted at a 1:20 (v/v) ratio in Hank’s balanced salt solution. For each examination, 5 µL of the diluted semen solution was placed on a CASA slide, which was then mounted on a microscope stage at 25 ^O^C. Sperm motility was activated by the addition of pond-filtered water, and sperm movements were recorded within one minute of activation under 200 × magnification. A total of six video fields were recorded, four tracks from the corners and 2 tracks from the center of the slide, following the protocol described by Alcántar‐Vázquez et al. (2022). The sperm velocity parameters, including curvilinear velocity (VCL, µm s ^−1^), defined as the average velocity along the actual curvilinear trajectory; straight-line velocity (VSL, µm s^−1^), the average velocity along a straight line between the first and last recorded positions; and average path velocity (VAP, µm s^−1^), representing the velocity along a smoothed average path (Boyers et al. 1989; Kime et al. 2001). Spermatozoa exhibiting a VAP greater than 5 μm/s were considered as motile (Alcántar‐Vázquez et al. 2022).

**Table 2:** Milt volume, pH, sperm concentration, motility, and velocity parameters

Items	Control group ± SD	ZnO-K group ± SD	CEO group ± SD	PSO group ± SD	*P*-value
Volume (ml/male)	**1.20**^**b**^** ± 0.052**	**1.40**^**a**^** ± 0.10**	**0.90**^**c**^** ± 0.054**	**0.80**^**c**^** ± 0.026**	**0.00**
pH	**6.700**^**a**^** ± 0.026**	**7.125**^**a**^** ± 0.025**	**7.000**^**a**^** ± 0.022**	**7.00**^**a**^** ± 0.018**	**0.09**
Sperm concentration (sperms ml^−1^ milt)	**5.285 × 10**^**9 a**^** ± 1.133**	**5.676 × 10**^**9 a**^** ± 0.879**	**5.540 × 10**^**9 a**^** ± 1.325**	**3.844 × 10**^**9 a**^** ± 0.742**	**0.59**
% Motility	**48.03**^**b**^** ± 7.26**	**66.21**^**a**^** ± 6.09**	**42.36**^**b**^** ± 4.68**	**38.99**^**b**^** ± 3.07**	**0.01**
Velocity (µm sec^−1^)					
VCL	**24.49**^**b**^** ± 0.677**	**43.95**^**a**^** ± 0.89**	**28.07**^**b**^** ± 0.751**	**25.03**^**b**^** ± 0.327**	**0.02**
VSL	**21.72**^**b**^** ± 1.313**	**32.19**^**a**^** ± 1.90**	**23.35**^**ab**^** ± 1.841**	**20.88**^**b**^** ± 0.838**	**0.05**
VAP	**20.94**^**b**^** ± 0.899**	**31.36**^**a**^** ± 1.036**	**20.19**^**b**^** ± 0.498**	**18.48**^**c**^** ± 0.318**	**0.02**

Values in the same row with different superscripts are significantly different (*P* < 0.05)

### Sperm vitality assessment

Sperm vitality was assessed by evaluating plasma membrane integrity using eosin-nigrosin staining (Kledmanee et al. 2013). Fresh milt samples, from the three replicates per experimental group, were diluted at a ratio of 1:49 (v/v) with 0.9% NaCl solution. Subsequently, 5 µl of diluted milt was rapidly mixed with 5 µl of 0.5% Eosin Y solution for 30 s, followed by the addition of 10% Nigrosin stain. A total of 200 spermatozoa per sample were examined under a light microscope at 400 × magnification. Sperm cells with intact plasma membranes remained unstained (indicating viability), while non-viable spermatozoa with compromised membranes absorbed the stain and appeared colored. The numbers and percentages of live and dead sperms were quantified using the CASA system.

### Spermatozoa DNA integrity

Sperm DNA integrity was evaluated in three milt sample replicates per experimental group using the sperm chromatin dispersion (SCD) assay, also known as the Halo Test. The procedure was based on the manufacturer's protocol (BASO Biotech Co. Ltd., Lot M22606, Taiwan) with minor modifications. Fresh milt was diluted in phosphate-buffered saline (pH 7.4), following the method described by Bombardelli et al. (2023). An aliquot of 7 μL of diluted milt was mixed with 120 μL of 0.5% low-melting-point agarose at 37 ^◦^C and spread evenly onto microscope slides pre-coated with 1.5% agarose. The slides were then treated with a lysis solution containing high salt and detergents to remove nuclear proteins and facilitate chromatin relaxation. Next, the slides were incubated with a denaturation solution containing 300 mM NaOH and 200 mM EDTA to induce DNA denaturation in spermatozoa with fragmented DNA. Following denaturation, the slides were washed, dehydrated, and stained with DNA-specific dyes provided in the kit. Sperm DNA integrity was examined under 400 × magnification and analyzed using the CASA system. Spermatozoa with intact DNA displayed characteristic halos of dispersed chromatin, whereas those with fragmented DNA lacked such halos.

### Sperm morphometric measurements

Fresh milt samples from each group were analyzed in triplicate. Each sample was diluted at a ratio of 1:49 (v/v) using a saline solution. A sperm smear was prepared by carefully spreading 5 µL of the diluted sample onto a clean microscope slide. The smears were then fixed with methanol and stained using eosin and methylene blue. Sperm morphometric measurements, including head length, head width, midpiece width, and tail length, were performed under 1000 × magnification, following the protocol described by Musa (2010). For each milt sample, 200 sperm cells were measured. Sperm abnormalities, including deviations in head and midpiece diameters as well as tail length, were described according to the criteria proposed by Paulino et al. (2016).

### Enzyme activity assays

Catalase (CAT) activity was determined using a commercial assay kit (Solarbio Life Sciences, Cat No. BC0200, China) following the manufacturer’s instructions. The assay was performed using 100 µL of the milt sample. The reaction was initiated by adding hydrogen peroxide (H₂O₂) to the sample, and CAT activity was assessed by monitoring the decrease in absorbance at 240 nm, corresponding to the decomposition of H₂O₂. Enzyme activity was expressed in units per milliliter (U/mL) of milt. Glutathione peroxidase (GPX) activity was measured using a GSH-PX assay kit (Elabscience, Cat. No. E-BC-K096-S, USA) according to the manufacturer’s protocol. The assay was conducted in a 100 µL milt sample and is based on the oxidation of reduced glutathione (GSH) in the presence of cumene hydroperoxide. The decrease in absorbance at 340 nm, reflecting NADPH consumption, was used to quantify GPX activity, which was expressed as milliunits per milliliter (mU/mL) of milt. Superoxide dismutase (SOD) activity was evaluated using a SOD assay kit (Solarbio Life Sciences, lot no. 809G113, China). The assay employed 100 µL of milt and is based on the enzyme’s ability to inhibit the reduction of nitroblue tetrazolium (NBT) by superoxide radicals. Absorbance was measured at 560 nm, and the activity was reported as U/mL of milt. All assays were conducted in triplicate to ensure reproducibility and accuracy. Negative controls (reaction mixtures without enzyme) and positive controls (standard enzyme solutions) were included in each assay batch to validate the results.

### Gene expression analyses

Approximately 25 µL of milt per sample, with three replicates from each group, were used for RNA isolation using TRIzol reagent (easy-RED, iNtRON Biotechnology, SKU:17,063, Korea), following the protocol of Heidary and Pahlevan (2014). Complementary DNA (cDNA) was synthesized from the extracted RNA using TOPscript™ RT-PCR DryMIX (Enzynomics, RT411, Korea), according to the method described by Mohammady et al. (2021). Quantitative real-time PCR (qPCR) was performed to quantify the expression of the *HSP70* and *CC chemokine* genes using gene-specific primers and SYBR™ Green Universal Master Mix (Applied Biosystems, cat. no. 4309155, USA). The primer sequences for amplification of *HSP70* were: forward 5'-CTCCACCCGAATCCCCAAAA-3'and reverse 5'-TCGATACCCAGGGACAGAGG-3'(Hassan et al. 2017). For *CC chemokine* amplification, the primers used were: forward 5'-ACAGAGCCGATCTTGGGTTACTTG-3'and reverse 5'-TGAAGGAGAGGCGGTGGATGTTAT-3'(Nakharuthai et al. 2016). The housekeeping gene *β-actin* was used as an internal reference, with the primers: forward (5'-TGGCAATGAGAGGTTCCG-3'and reverse 5'-TGCTGTTGTAGGTGGTTTCG-3'(Tanomman et al. 2013). Gene expression levels were normalized using the 2^−ΔΔCt^ method (Livak and Schmittgen 2001). PCR amplification was conducted on an Applied Biosystems 7500 Real-Time PCR System. The thermal cycling conditions included an initial denaturation at 95 °C for 10 min, followed by 40 cycles of denaturation at 95 °C for 10 s, annealing at 60 °C for *CC chemokine* and 64 °C for *HSP70* for 30 s, and extension at 72 °C for 30 s. A final extension step was performed at 72 °C for 10 min. Melting curve analysis was conducted at the end of the amplification to confirm the specificity of the PCR products. Gene expression data are presented as fold changes relative to the control group (basic diet).

### Statistical analyses

Statistical analyses were conducted using SAS software (version 9.22; SAS Institute Inc., Cary, NC, USA). Prior to analysis, data were tested for normality and homogeneity of variances. Descriptive statistics and one-way analysis of variance (ANOVA) were used to evaluate differences among groups. When significant effects were detected, Duncan’s multiple range test was applied for comparisons. Results were presented as mean ± standard deviation (SD), and statistical significance was defined as *P* < 0.05.

Gene expression data were analyzed in RStudio (version 2023.3.0.386) using the ggplot2, dplyr, and tidyverse packages (Wickham and Grolemund 2016). One-way ANOVA was performed to assess differences in gene expression among groups, followed by Tukey’s honest significant difference (HSD) test for multiple comparisons. Gene expression results were reported as mean ± standard error (SE) to reflect variability within groups.

## Results

### The physical characteristics of milt

Table 2 presents the milt volume, pH, and sperm concentration across the different treatment groups. The ZnO-K group exhibited the highest milt volume (1.40 ± 0.10 ml/male), which was significantly greater than that of the other groups. In contrast, the PSO group recorded the lowest milt volume (0.80 ± 0.09 ml/male). Milt pH values ranged from 6.700 ± 0.026 in the control group to 7.125 ± 0.025 in the ZnO-K group. The ZnO-K group also demonstrated the highest sperm concentration (5.676 X 10^9^ sperm/ml), followed closely by the CEO group (5.540 X 10^9^ sperm/ml). Although the PSO group had the lowest sperm concentration (3.844 × 10⁹ sperm/ml), the differences in sperm concentration among groups were not statistically significant (Table 2).

### Sperm motility and velocity

The ZnO-K group demonstrated the highest percentage of motile spermatozoa (66.21%), which was significantly greater than that of all other groups. In contrast, the PSO group exhibited the lowest motility (38.99%). ZnO-K supplementation also significantly enhanced sperm velocity parameters, with curvilinear velocity (VCL) of 43.95 ± 0.89 µm/s and an average path velocity (VAP) of 31.36 ± 1.04 µm/s (Table 2). Although the straight-line velocity (VSL) in the ZnO-K group (32.19 ± 1.90 µm/s) was higher than that in the CEO group (23.35 ± 1.84 µm/s), the difference was not statistically significant. Moreover, no significant differences in VCL and VSL were observed among the control, CEO, and PSO groups.

### Sperm vitality, DNA integrity, and morphological characteristics

The percentage of live spermatozoa, as indicated by intact plasma membranes, was significantly higher in the ZnO-K (65.29% ± 0.55) and CEO (70.78% ± 4.42) groups compared to the other groups (Table 3).

**Table 3:** Sperm vitality, DNA integrity, and morphometric measurements

Items	Control group ± SD	ZnO-K group ± SD	CEO group ± SD	PSO group ± SD	*P*-value
% Live sperms	**49.97**^**b**^** ± 0.74**	**65.29**^**a**^** ± 0.55**	**70.78**^**a**^** ± 4.42**	**41.14**^**c**^** ± 1.32**	**0.00**
% DNA integrity	**28.08**^**b**^** ± 6.881**	**60.08**^**a**^** ± 1.714**	**54.23**^**a**^** ± 6.729**	**34.88**^**b**^** ± 7.593**	**0.00**
Morphological measurements (µm)					
Head length	**2.82**^**a**^** ± 0.09**	**2.56**^**a**^** ± 0.11**	**2.41**^**a**^** ± 0.13**	**3.08**^**a**^** ± 0.31**	**0.08**
Head width	**2.15**^**ab**^** ± 0.15**	**1.80**^**b**^** ± 0.13**	**1.84**^**b**^** ± 0.10**	**2.56**^**a**^** ± 0.30**	**0.03**
Midpiece width	**0.57**^**a**^** ± 0.040**	**0.66**^**a**^** ± 0.05**	**0.59**^**a**^** ± 0.05**	**0.51**^**a**^** ± 0.06**	**0.25**
Tail length	**18.83**^**a**^** ± 0.46**	**19.66**^**a**^** ± 0.57**	**17.87**^**b**^** ± 0.65**	**14.92**^**c**^** ± 0.52**	**0.00**

Values in the same row with different superscripts are significantly different (*P* < 0.05)

**Fig. 1:**
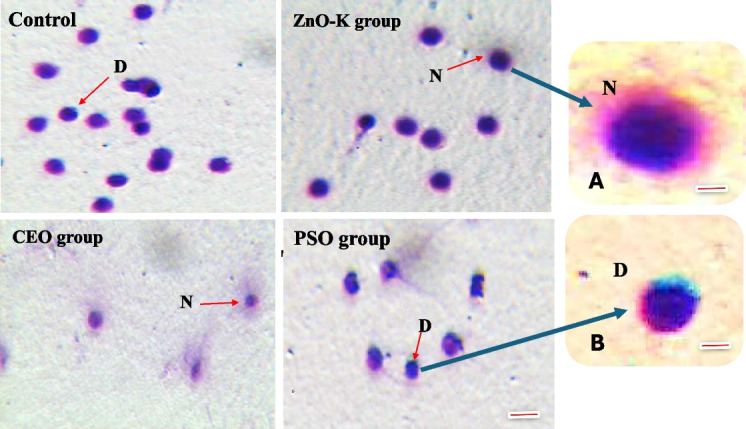
CASA for sperm DNA integrity. **A** and **B**, enlargements of sperm heads. N, the head of sperm shows non-fragmented DNA, with a halo. D, head of sperm shows fragmented DNA, without halo. Scale bar = 5 µm; bar in A or B = 1.5 µm

DNA integrity analysis (Table 3 and Fig. 1) showed that spermatozoa from the ZnO-K and CEO groups had the highest percentages of intact DNA, at 60.08% ± 1.741 and 54.23% ± 6.729, respectively. These values were significantly greater than those observed in the control (28.08% ± 6.88) and PSO (34.88% ± 7.59) groups.

**Fig. 2:**
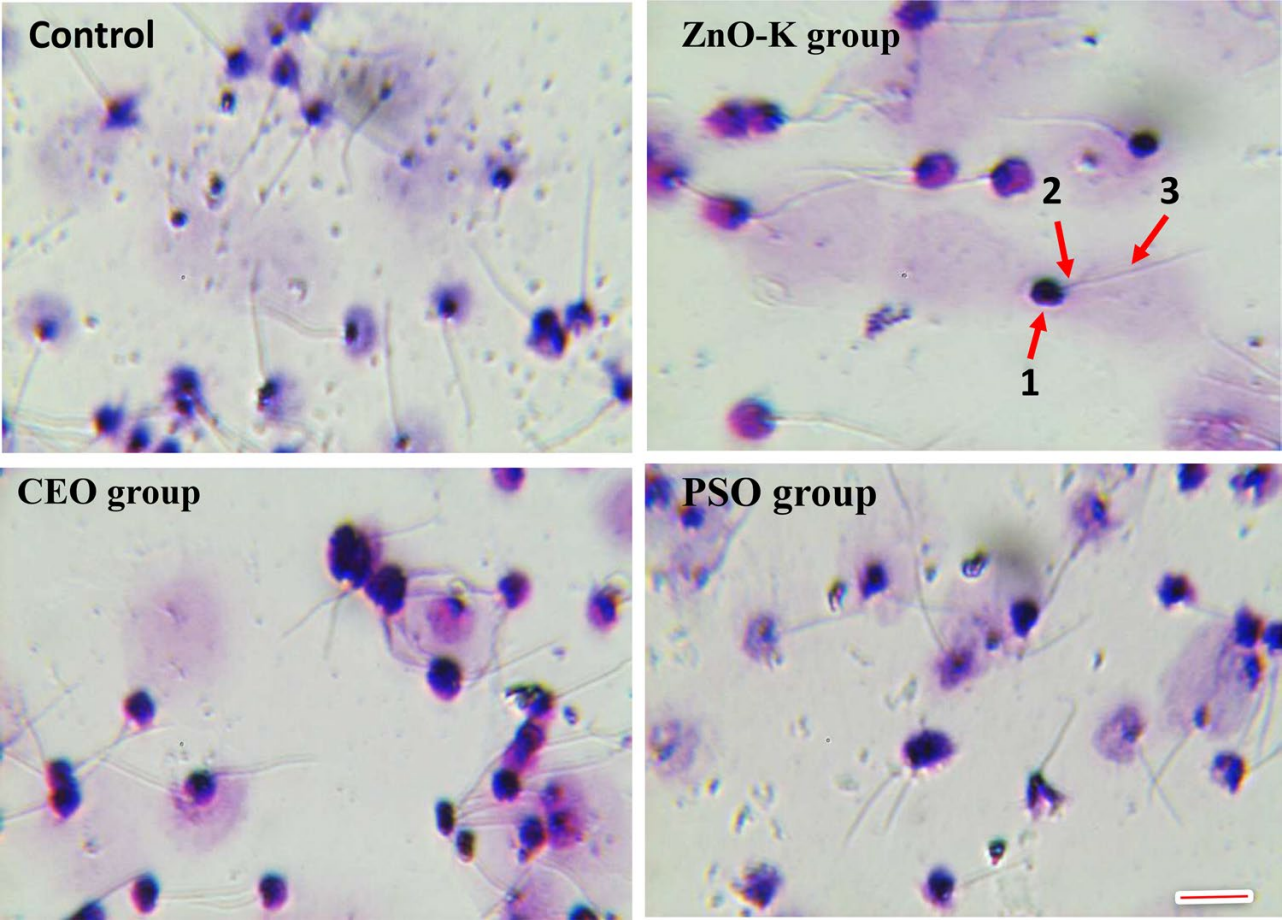
Sperm morphological structures for males fed on diets supplemented with different antioxidants, 1. Head sperm, 2. Midpiece and 3. Tail. Scale bar = 10 µm

Morphological assessment revealed that the sperm heads were spherical across all groups, with significant differences in head width. The average head widths were 2.15 ± 0.15 µm in the control group, 1.08 ± 0.13 µm in the ZnO-K group, 1.84 ± 0.10 µm in the CEO group, and 2.56 ± 0.30 µm in the PSO group. Additionally, the ZnO-K group exhibited the longest sperm tails (19.66 ± 0.57 µm) (Fig. 2). In contrast, the PSO group showed spermatozoa with relatively wider heads and shorter tails compared to the other groups (Table 3; Fig. 2).

### Effect of dietary supplements on enzymatic activities in sperm

The activities of the antioxidant enzymes CAT, GPX, and SOD are presented in Table 4. The ZnO-K group exhibited a significantly higher CAT activity (47.33 ± 1.452 U/ml), compared to the control group (32.67 ± 2.603 U/ml) and all other treatment groups (*P* < 0.05). Similarly, GPX activity was significantly elevated in the ZnO-K group (65.67 ± 5.547 mU/ml), relative to CEO group (41.67 ± 1.763 mU/ml) and the PSO group (32.00 ± 2.081 mU/ml). In addition, the ZnO-K group showed a significantly higher SOD activity (60.67 ± 3.382 U/ml), compared to all other groups.

**Table 4:** Enzymatic activities in sperms of *O. niloticus* males

Items	Control group ± SD	ZnO-K group ± SD	CEO group ± SD	PSO group ± SD	*P*-value
CAT (U/ml)	**32.67**^**b**^** ± 2.603**	**47.33**^**a**^** ± 1.452**	**28.00**^**bc**^** ± 1.527**	**24.00**^**c**^** ± 2.886**	**0.00**
GPX (mU/ml)	**48.00**^**b**^** ± 4.582**	**65.67**^**a**^** ± 5.547**	**41.67**^**bc**^** ± 1.763**	**32.00**^**c**^** ± 2.081**	**0.00**
SOD (U/ml)	**40.00**^**b**^** ± 4.041**	**60.67**^**a**^** ± 3.382**	**43.00**^**b**^** ± 3.511**	**36.67**^**b**^** ± 3.179**	**0.01**

Values in the same row with different superscripts are significantly different (*P* < 0.05). CAT: Catalase (U/ml) as unit per milliliter; GPX: Glutathione peroxidase mU/ml) as milliunit per milliliter; SOD: Superoxide dismutase (U/ml) as unit per milliliter

### Effect of dietary supplements on gene expression in sperm

**Fig. 3:**
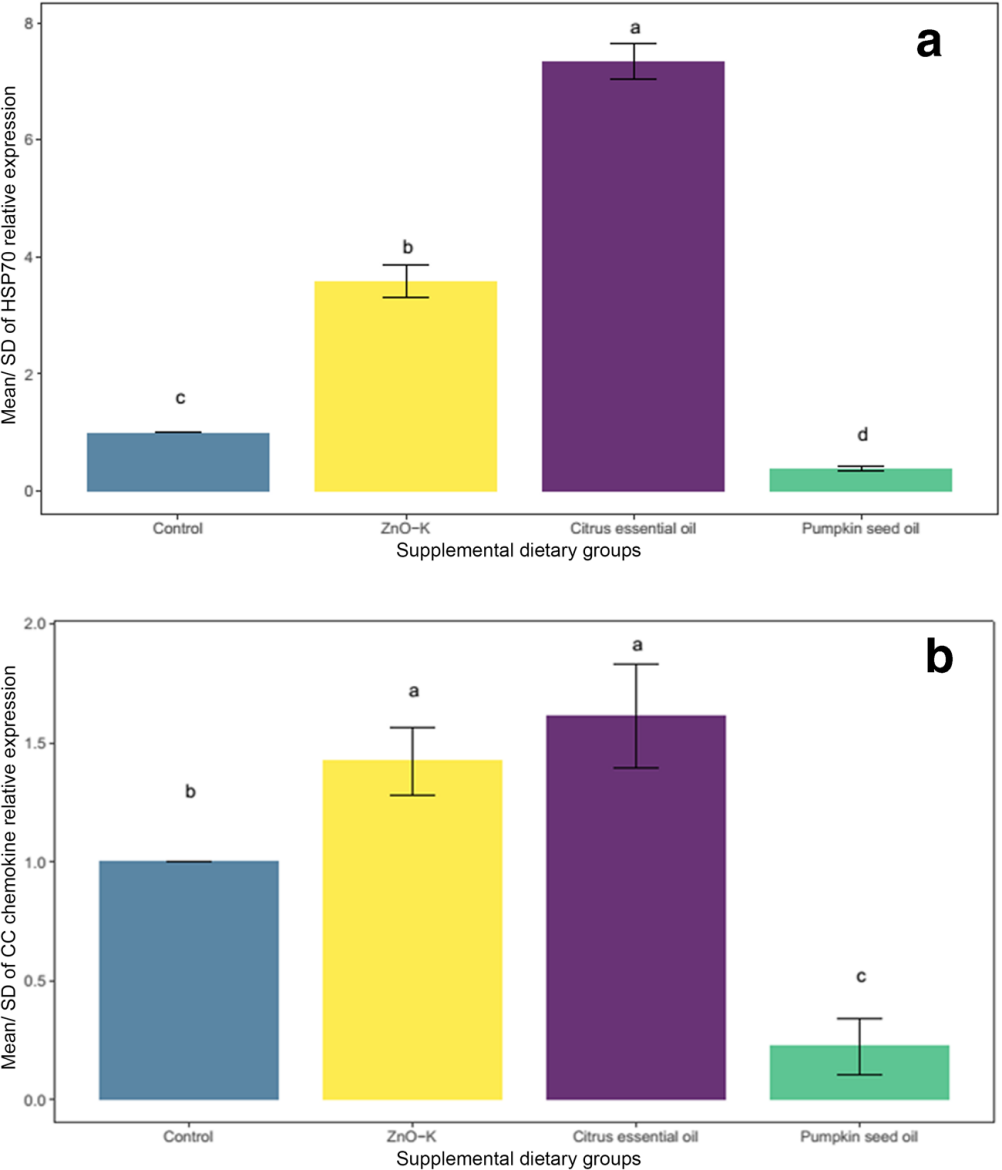
**a** Effect of the supplements ZnO-K, citrus essential oil, and pumpkin seed oil on the gene HSP70 expression in the sperms of O. niloticus. Bars with different superscript letters (a, b, c, d) illustrate significant differences (p < 0.05). Error bars +/- 1 SD. **b** Effect of the supplements ZnO-K, citrus essential oil, and pumpkin seed oil on the gene CC chemokine expression in the sperms of O. niloticus. Bars with different superscript letters (a, b, c) illustrate significant differences (p < 0.05). Error bars +/- 1 SD

Quantitative PCR analysis demonstrated a significant increase in *HSP70* expression in sperm samples from the CEO and ZnO-K groups (Fig. 3a). Among these, the CEO group exhibited a significantly higher level of *HSP70* expression (*P* < 0.05), compared to the ZnO-K group, as confirmed by Tukey’s post-hoc test. In contrast, the PSO treatment led to a significant reduction in *HSP70* expression relative to all other groups (Fig. 3a).

Similarly, *CC chemokine* expression was significantly upregulated in the CEO and ZnO-K groups compared to the control (Fig. 3b). However, no significant difference was observed between the CEO and ZnO-K treatments. Tukey's post-hoc analysis confirmed that PSO supplementation resulted in a significant downregulation of *CC chemokine* expression compared to all other groups.

## Discussion

This study investigated the effects of diet supplements, ZnO-K, CEO, and PSO, on sperm quality in *O. niloticus*. Integration of advanced tools, including CASA parameters, DNA integrity assays, enzymatic activity measurements, and gene expression profiling, validated sperm examination, and introduced a robust sperm quality assessment under the effect of the studied dietary supplements.

To minimize the potential confounding effects of individual variability on the outcomes of the dietary supplementation, we carefully selected male tilapia with almost having same morphometric (e.g., body length, weight, gonadosomatic index) and physiological traits, which positively correlated with both the quantity and quality of sperm produced by an individual (Bhujel 2000; Mylonas et al. 2010; El-Greisy and El-Gamal 2012; Kongbo et al. 2024). This standardization was implemented to ensure that all experimental treatments had a similar baseline condition optimized for healthy sperm production. By controlling for individual differences, we aimed to attribute any observed variations in sperm quality more reliably to the administered dietary supplements, rather than to inherent biological differences among individuals.

The ZnO-K treatment resulted in a significant increase in both milt volume and sperm concentration. Remarkably, the milt volume per male in this group exceeded values previously reported for *O. niloticus* fed diets supplemented with *Tribulus terrestis* extract and 17α-methyl testosterone (Hassona et al. 2020), as well as those fed diets containing *Spirulima platensis*, as a feed additive (El-daim et al. 2021). Zinc plays a critical role in promoting spermatogenesis in fish (Yamaguchi et al. 2009). Although the CEO group also exhibited relatively high milt volume per male, CEO is composed of up to 90% limonene, a compound with established antioxidant properties that can enhance sperm quality (Eddin et al. 2021). Nevertheless, this study represents the first evaluation of CEO's effects on sperm health in Nile tilapia, and further investigations are needed to elucidate its underlying mechanisms of action. The milt pH remained stable across all treatments, consistent with the findings of Ahamed et al. (2020), who reported no dietary influence on milt pH in *O. mossambicus*.

Sperm motility and velocity are critical parameters for evaluating semen quality (Gallego and Asturiano 2019). The observed sperm velocity values in the ZnO-K group were within the typical range reported for healthy sperm in Nile tilapia (Gennotte et al. 2012; Dzyuba et al. 2019; Su et al. 2024). The CEO group exhibited significantly lower sperm velocity values compared to the ZnO-K group. This reduction may be attributed to the presence of citrus-derived monoterpenoid aldehyde isomers, such as geranial and neral, which are known to impair mitochondrial function and consequently reduce sperm velocity parameters (Cavalleri et al. 2018). Comparable reductions in sperm motility were reported in mice exposed to citrus lemon oil (Rahayu and Hanizar 2021). Among all treated groups, the PSO group exhibited the lowest sperm velocity values. This may be due to the presence of anti-nutritional factors, such as phytates, which may chelate essential minerals required for sperm motility (Elinge et al. 2012; Nayyef et al. 2023). Overall, the sperm velocity values recorded in this study differed from those reported by Sarmento et al. (2017), likely due to differences in dietary supplementation, feeding durations, and methodologies used for sperm motility assessment. Additionally, a notable discrepancy was observed when comparing the current findings to those of Bombardelli et al. (2023), who reported higher sperm velocities in genetically improved farmed tilapia. This variation may be attributed to the use of wild broodstock in the present study. These inter-study differences underscore the need for a standardized framework for the measurement of sperm velocity in fish. Standardization would enhance the reliability and reproducibility of results, facilitate cross-study comparisons, and support the development of validated protocols for sperm quality assessment (Blackburn et al. 2022). In this study, complementary assessments, including DNA integrity, antioxidant enzyme activity, and gene expression analysis, provided further insights into the overall evaluation of sperm quality.

The improved plasma membrane integrity observed in the ZnO-K group may be explained by the upregulation of antioxidant enzymes (CAT, GPX, SOD), which are essential for protecting sperm from oxidative stress and maintaining functional integrity (Soaudy et al. 2021). Additionally, the bactericidal properties of CEO (Li et al. 2018) may have contributed to preserving membrane integrity. Notably, sperm from the ZnO-K group exhibited longer tails, which were positively associated with increased sperm velocity, in agreement with previous findings in *Barbus* species (Alavi et al. 2008). Moreover, the relationship between sperm midpiece size and mitochondrial sheath length is crucial for motility, as it determines the energy-generating capacity of sperm cells (Morita et al. 2004). Zinc has been shown to influence the structural optimization of these components, thereby enhancing sperm performance (Arruda et al. 2021).

DNA integrity, a key indicator of sperm quality, was highest in the ZnO-K group, likely due to Zinc’s role in chromatin stabilization and oxidative damage mitigation (Björndahl and Kvist 2011; Huang et al. 2021). The presence of fragmented DNA in the control group may reflect unidentified sources of oxidative stress, a phenomenon previously observed in control-group *O. niloticus* (Bombardelli et al. 2023). Due to the limited cytoplasmic content of fish spermatozoa, which results in low antioxidant reserves, these cells are particularly susceptible to oxidative damage (Cosson 2019; Cabrita et al. 2014).

Sperm from the ZnO-K group exhibited significantly higher activities of CAT, GPX, and SOD activities, underscoring the role of zinc in enhancing antioxidant defense mechanisms. Zinc serves as a critical cofactor for numerous antioxidant enzymes, thereby mitigating oxidative stress and contributing to improved reproductive health (Fallah et al. 2018). These findings were consistent with previous reports demonstrating the involvement of SOD and GPX in preventing oxidative stress-induced sperm damage across various fish species (Shaliutina-Kolešová et al. 2018). Moreover, zinc supplementation has been shown to upregulate the synthesis of zinc-dependent antioxidant enzymes, leading to improved sperm quality (Cheng and Chen 2021). In the current investigation, the elevated enzyme activities were associated with enhanced sperm motility and DNA integrity in the ZnO-K group, supporting the positive effects of zinc on multiple sperm quality parameters.

The current study demonstrated that dietary supplementation with CEO significantly elevated the expression of *HSP70* and *CC chemokine* in the spermatozoa of *O. niloticus*, suggesting a potent role for CEO in enhancing cellular stress resilience and immune modulation at the molecular level. HSP70, a highly conserved member of the heat shock protein family, plays a critical role in cellular defense by stabilizing nascent peptides, preventing protein aggregation, and assisting in protein folding, especially under stress conditions such as oxidative damage or temperature fluctuations (Kiang and Tsokos 1998; Lindquist and Craig 1988). The marked upregulation of *HSP70* in the CEO group, exceeding even the expression levels in ZnO-K-treated fish, underscored the strong cytoprotective potential of CEO. This may be attributed to its rich composition of bioactive compounds, such as limonene, linalool, and citral, known for their antioxidant and membrane-stabilizing properties (Ben Hsouna et al. 2017). Hence, the elevated *HSP70* expression suggests an enhanced ability of sperm cells to withstand environmental and oxidative stress during handling and fertilization, a critical advantage in artificial reproduction and cryopreservation protocols. Likewise, the significant upregulation of *CC chemokine* expression in CEO-treated males suggests an immunomodulatory effect of citrus-derived compounds on the male reproductive system. CEO supplementation may influence immune pathways indirectly by modulating the testicular microenvironment, reducing oxidative stress, and promoting the integrity of sperm cells. These findings were consistent with previous studies reporting that essential oils from citrus enhance antioxidant defense mechanisms and modulate stress-responsive gene expression in aquatic species (Souza et al. 2019). The dual role of CEO-promoting antioxidant stability through *HSP70* and supporting immune readiness via *CC chemokine* upregulation positions it as a promising phytogenic additive for improving sperm quality. However, this study added basic knowledge about the relationship between sperm quality and the expression levels of the genes, *HSP70,* and *CC chemokine*, supporting the crucial role of ZnO-K and CEO, as antioxidant key players, in enhancing the expression of immune response genes in crayfish, *Procambarus clarkii* (Kong et al. 2024), and zebrafish, *Danio rerio* (Mahjoubian et al. 2023). Further investigation is warranted to identify the precise molecular targets and signaling pathways activated by ZnO-K and CEO constituents. Integrative studies involving testicular transcriptomics, proteomics, and functional sperm assays could provide deeper insights into CEO’s role in reproductive physiology and its potential as a tool for improving sperm cryotolerance and fertility outcomes in tilapia hatcheries. On the other hand, limited knowledge is known about the mechanisms of the negative effect of PSO on the expression of *HSP70* and *CC chemokine* in tilapia sperm, and further studies should be done to clarify the suppression of these gene expressions by current PSO supplementation.

In conclusion, the present study demonstrated the potential of ZnO-K as a dietary supplement for enhancing sperm quality in *O. niloticus*. The observed improvements are likely attributed to its multifaceted role in enhancing sperm motility and velocity, preserving DNA integrity, strengthening antioxidant defense mechanisms, and upregulating the expression of immunity-related genes. Additionally, CEO exhibited positive effects on sperm parameters, including concentration, velocity, viability, DNA integrity, and the upregulation of *HSP70* and *CC chemokine* gene expression. In contrast, PSO, showed the least efficacy, suggesting the need for further optimization or potential combination with other supplements to maximize its potential benefits in improving sperm quality. Thus, the current study highlighted critical parameters for evaluating sperm quality in tilapia, offering valuable insights into factors that directly influence the outcomes of artificial fertilization in hatchery environments. By systematically assessing aspects such as motility, viability, morphology, DNA integrity, antioxidant enzymes, and sperm immunogen expression, under current food additives, the research established a framework for standardized sperm quality assessment. These findings not only contribute to improving fertilization success rates in aquaculture operations but also strengthen the foundational criteria for the development and application of effective sperm biobanking protocols. Ultimately, the integration of these quality indicators into routine practices can enhance genetic resource conservation, support selective breeding programs, and promote sustainable tilapia aquaculture.

## Supplementary Information

Below is the link to the electronic supplementary material.

Supplementary file1 (DOCX 16 KB)

## Data Availability

No datasets were generated or analysed during the current study.

## Declarations

### Authentication

The experimental fish handling protocol was approved by the National Institute of Oceanography and Fisheries (NIOF) committee for institutional care of aquatic organisms and experimental animals, under the no. NIOFAQ5F25R009.
