# Supplementary material for: Ameliorative potential of dietary supplements, ZnO-K, citrus essential oil, and pumpkin seed oil, on sperm quality in Nile tilapia: Insights from CASA, DNA integrity, antioxidant enzymes, and gene expressions
Source: Fish Physiol Biochem. 2025 Jun 23;51(4):114. doi: 10.1007/s10695-025-01529-4 (PMC12185577; doi:10.1007/s10695-025-01529-4)
Supplement: Supplementary file 1 — Supplementary file1 (DOCX 16 KB) [file 10695_2025_1529_MOESM1_ESM.docx]

Supplementary Table 1: Chemical composition of citrus essential oil (CEO)*

| Components | % |
| --- | --- |
| α-Pinene | 0.46 |
| Sabinene | 0.44 |
| β-Pinene | 25.44 |
| Myrcene | 0.36 |
| Limonene | 39.74 |
| 1,8-cineol | 0.54 |
| cis-linalool oxide | 0.53 |
| trans-linalool oxide | 0.49 |
| Linalool | 2.16 |
| Terpinen-4-ol | 0.26 |
| Myrtenal | 0.43 |
| α-Terpineol | 7.30 |
| Nerol | 0.99 |
| linalyl acetate | 3.01 |
| Geranial | 0.43 |
| Indol | 0.45 |
| anthraniate méthyl | 0.66 |
| Acetate neryl | 1.74 |
| Acétate geranyl | 3.03 |
| Nerolidol | 6.91 |
| Farnesol | 4.28 |
| Monoterpene hydrocarbon | 67.86 |
| Oxygenated monoterpenes  Nitrogen components | 30.68  1.11 |
| Total % | 99.65 |

*(Ben Hsouna et al. 2017)

Supplementary Table 2: Fatty acid (%) and phenolic acids (mg/100 g) composition of pumpkin seed oil (PSO)

| Fatty acids |  |
| --- | --- |
| Palmitic (C16:0)  Palmitoleic (C16:1) | 15.97± 0.39  tr. |
|  |  |
| Stearic (C18:0) | 4.68±0.56 |
| Oleic (C18:1) | 44.11±0.63 |
| Linoleic (C18:2) | 34.77±0.95 |
| Linolenic (C18:3) | tr. |
| Arachidic (C20:0) | 0.41±0.40 |
| SAFA | 21.07±1.19 |
| MUFA | 44.12±0.57 |
| PUFA | 34.78±0.85 |
| Phenolic acids |  |
| Protocatechuic acid | 1.81 ± 0.26 |
| Caffeic acid | 3.88± 0.03 |
| Syringic acid | 7.96±0.13 |
| Vanillic acid | 2.46±0.37 |
| p-coumaric acid | 2.50±0.95 |
| Ferulic acid | 4.99±0.29 |

tr.: trace amounts (less than 0.2%). Values are means ± SD of three determinations (Rezig et al. 2012)
